# Small RNA interference-mediated gene silencing of heparanase abolishes the invasion, metastasis and angiogenesis of gastric cancer cells

**DOI:** 10.1186/1471-2407-10-33

**Published:** 2010-02-05

**Authors:** Liduan Zheng, Guosong Jiang, Hong Mei, Jiarui Pu, Jihua Dong, Xiaohua Hou, Qiangsong Tong

**Affiliations:** 1 Department of Pathology, Union Hospital of Tongji Medical College, Huazhong University of Science and Technology, Wuhan 430022, Hubei Province, China; 2 Department of Surgery, Union Hospital of Tongji Medical College, Huazhong University of Science and Technology, Wuhan 430022, Hubei Province, China; 3 Department of Central Laboratory, Union Hospital of Tongji Medical College, Huazhong University of Science and Technology, Wuhan 430022, Hubei Province, China; 4 Department of Gastroenterology, Union Hospital of Tongji Medical College, Huazhong University of Science and Technology, Wuhan 430022, Hubei Province, China

## Abstract

**Background:**

Heparanase facilitates the invasion and metastasis of cancer cells, and is over-expressed in many kinds of malignancies. Our studies indicated that heparanase was frequently expressed in advanced gastric cancers. The aim of this study is to determine whether silencing of heparanase expression can abolish the malignant characteristics of gastric cancer cells.

**Methods:**

Three heparanase-specific small interfering RNA (siRNAs) were designed, synthesized, and transfected into cultured gastric cancer cell line SGC-7901. Heparanase expression was measured by RT-PCR, real-time quantitative PCR and Western blot. Cell proliferation was detected by MTT colorimetry and colony formation assay. The *in vitro *invasion and metastasis of cancer cells were measured by cell adhesion assay, scratch assay and matrigel invasion assay. The angiogenesis capabilities of cancer cells were measured by tube formation of endothelial cells.

**Results:**

Transfection of siRNA against 1496-1514 bp of encoding regions resulted in reduced expression of heparanase, which started at 24 hrs and lasted for 120 hrs post-transfection. The siRNA-mediated silencing of heparanase suppressed the cellular proliferation of SGC-7901 cells. In addition, the *in vitro *invasion and metastasis of cancer cells were attenuated after knock-down of heparanase. Moreover, transfection of heparanase-specific siRNA attenuated the *in vitro *angiogenesis of cancer cells in a dose-dependent manner.

**Conclusions:**

These results demonstrated that gene silencing of heparanase can efficiently abolish the proliferation, invasion, metastasis and angiogenesis of human gastric cancer cells *in vitro*, suggesting that heparanase-specific siRNA is of potential values as a novel therapeutic agent for human gastric cancer.

## Background

Gastric cancer is one of the most common cancer types in the world, although its incidence has gradually decreased in recent years in many countries [1]. Invasion and metastasis of cancer cells remains the main cause of gastric cancer-related death [2]. It is well known that the basement membrane (BM) and extracellular matrix (ECM) play a barrier to prevent tumor cells from invasion and metastasis [3]. Specific enzymes produced by cancer cells and activated by certain signals, such as matrix metalloproteinases (MMPs) and urokinase-type plasminogen activator (uPA), have been reported to degrade BM and ECM, and are associated with progression of gastric cancer [4-7]. A better knowledge of changes in gene expression during invasion and metastasis may lead to improvements in the treatment of advanced gastric cancer.

Heparan sulfate (HS) and heparin sulfate proteoglycans (HSPGs), the important structural components of ECM and external surface of cell membranes, play a major role in cell-cell and cell-ECM interactions [8]. Previous reports have shown that heparanase (HPA), an endo-h-D-glucuronidase, has the ability to cleave the heparan sulfate chain of HSPGs, and is one of the key enzymes involved in the invasion and metastasis of malignant tumors [9]. Under normal physiological conditions, HPA expression is detectable in endothelial cells, smooth muscle cells, cytotrophoblasts, keratinocytes, platelets platelets, neutrophils, and activated T lymphocytes [10]. However, HPA expression is up-regulated in many tumor cells including esophageal carcinoma, pancreatic carcinoma, melanoma, bladder cancer, and prostate cancer [11-15]. It has been established that a significant correlation of HPA over-expression is coupled with increased metastatic potential and decreased survival rates of cancer [16]. These studies suggest that HPA is correlative with invasion and metastasis of cancer cells, and served as an important target of cancer therapy.

Previous studies indicate that the expression of HPA was frequently observed in advanced gastric cancers [17,18]. The frequency was significantly correlated with histopathological parameters reflecting invasive and metastatic potentials and prognosis of gastric cancers [17,18]. Our studies also demonstrated the over-expression of HPA protein in advanced gastric cancer (data not shown). However, it still remains largely unknown whether inhibiting HPA expression can abolish the invasion and metastasis of gastric cancer cells. Due to the non-specific activities of current HPA inhibitors and the great difficulties in identifying efficient inhibitors [19-22], genetic approaches targeting HPA have been regarded as a promising alternative [23]. RNA interference (RNAi) is a posttranscriptional mechanism of gene silencing through chromatin remodeling, inhibition of protein translation or direct mRNA degradation [24]. Chemically synthetic small interfering RNA (siRNA) is currently being evaluated not only as an extremely powerful instrument for functional genomic analyses, but also as a potentially useful method to develop highly specific gene-silencing therapeutics [25]. In this study, we designed HPA-specific siRNAs and evaluated their gene silencing effects in cultured gastric cancer cells. We demonstrated that silencing of HPA expression attenuated the *in vitro *invasion, metastasis and angiogenesis capabilities of gastric cancer cells.

## Methods

### Cell culture

Human gastric cancer cell line SGC-7901 and endothelial cell line HUVEC were purchased from American Type Culture Collection (ATCC) and grown in RPMI1640 medium (Life Technologies, Inc., Gaithersburg, MD) supplemented with 10% fetal bovine serum (FBS, Life Technologies, Inc.), penicillin (100 U/ml) and streptomycin (100 μg/ml). Cells were maintained at 37°C in a humidified atmosphere of 5% CO_2_.

### Heparanase-specific siRNA and transfection

For RNA interference, three 21-nucleotide siRNA duplexes targeting different encoding regions of HPA (GenBank accession No. AF165154) were designed and chemically synthesized (Ribobio Co. Ltd, GuangZhou, China). The secondary structures of target mRNA were predicted by RNAstructure 3.7 software [26]. The nucleotide sequences were as follows: siH1 (1088-1106 bp), 5'-UUAUGUGGCUGGAUAAAUUtt-3' (sense), and 5'-AAUUUAUCCAGCCACAUAAtt-3' (antisense); siH2 (1267-1285 bp), 5'-GUGCAAGGUUCAAAGAGAAtt-3' (sense), and 5'-UUCUCUUUGAACCUUGCACtt-3' (antisense); siH3 (1496-1514 bp), 5'-CUCUAAAGA UGGUGGAUGAtt-3' (sense), and 5'-UCAUCCACCAUCUUUAGAGtt-3' (antisense). The sequences 5'-AGCAUCGUACGUAGGCCAGtt-3' (sense), and 5'-CUGGCCUACGUACGAUG CUtt-3' (antisense) were used as a scrambled siRNA control (mock). The siRNAs were transfected into culture cells with Genesilencer Transfection Reagent (Genlantis, San Diego, CA), according to the manufacturer's instructions.

### Real-time quantitative PCR

Total RNA was isolated with RNeasy Mini Kit (Qiagen Inc., Valencia, CA). The reverse transcription reactions were conducted with Transcriptor First Strand cDNA Synthesis Kit (Roche, Indianapolis, IN). The PCR primers were designed by Premier Primer 5.0 software as the following: for human HPA 5'-GAATGGACGGACTGCTAC-3' and 5'-CCAAAGAATACTTGCC TCA-3' amplifying a 261-bp fragment; for human GAPDH 5'-AGAAGGCTGGGGCTCATT TG-3' and 5'-AGGGGCCATCCACAGTCTTC-3' amplifying a 258-bp fragment. Real-time PCR with SYBR Green PCR Master Mix (Applied Biosystems, Foster City, CA) was performed using ABI Prism 7700 Sequence Detector (Applied Biosystems). The fluorescent signals were collected during extension phase, Ct values of the sample were calculated, and HPA transcript levels were analyzed by 2^-ΔΔCt ^method.

### Western blot

Cellular protein was extracted with 1× cell lysis buffer (Promega, Madison, WI). Protein (50 μg) from each sample was subjected to 4-20% pre-cast polyacrylamide gel (Bio-Rad, Hercules, CA) electrophoresis and transferred to nitrocellulose membranes (Bio-Rad). For HPA (InSight Company, Rehovot, Israel) and GAPDH (Santa Cruz Biotechnology, Santa Cruz, CA) detection, the primary antibody dilutions were 1:500 and 1:1000, respectively, followed by 1:3000 dilution of goat anti-rabbit HRP-labeled antibody (Bio-Rad). ECL substrate kit (Amersham, Piscataway, NJ) was used for the chemiluminscent detection of signals with autoradiography film (Amersham).

### Measurement of cell viability

Forty-eight hrs post-transfection, cell viability was monitored by the 2-(4, 5-dimethyltriazol-2-yl)-2,5-diphenyl tetrazolium bromide (MTT, Sigma) colorimetric assay. Briefly, 20 μl of MTT (5 mg/ml) was added to each well. After 4 hrs of incubation at 37°C, the cell supernatants were discarded, MTT crystals were dissolved with DMSO and the absorbance measured at 570 nm. Percent viability was defined as the relative absorbance of transfected versus untransfected control cells. All experiments were done with 6-8 wells per experiment and repeated at least three times.

### Colony formation assay

Forty-eight hrs post-transfection, the cells were seeded at a density of 300/ml on 35-mm dishes. Colonies were allowed to grow for 10-14 days. The medium was discarded and each well was washed twice with phosphate buffered saline (PBS) carefully. The cells were fixed in methanol for 15 min, and then stained with crystal violet for 20 min. Finally, positive colony formation (more than 50 cells/colony) was counted. The survival fraction for cells was expressed as the ratio of plating efficiency of transfected cells to that of untransfected control cells.

### Cell adhesion assay

Forty-eight hrs post-transfection, 2 × 10^4 ^cells were inoculated into each well of 96-well plates that were precoated with 100 μl of 20 μg/ml matrigel (BD Biosciences, Franklin Lakes, NJ), and incubated at 37°C in serum-free complete medium (pH 7.2) for 2 hrs. After incubation, the wells were washed three times with PBS and the remaining cells were fixed in 4% paraformaldehyde for 20 min at room temperature. The cells were stained with 0.1% crystal violet and washed three times with PBS to remove free dye. After extraction with 10% acetic acid, absorbance of the samples was measured at 570 nm. 0%, 20%, 50% and 100% of inoculated cells were directly fixed in 4% paraformaldehyde 2 hrs after inoculation.

### Scratch migration assay

SGC-7901 cells were transfected in 24-well plate with siRNAs. Forty-eight hrs post-transfection, the cells were scraped with the fine end of 1-ml pipette tips (time 0). Plates were washed twice with PBS to remove detached cells, and incubated with the complete growth medium. Cell migration into the wounded empty space was followed after 24 hrs and photographed.

### Matrigel invasion assay

The Boyden chamber technique (transwell analysis) was performed. Briefly, the 8-μm pore size filters were coated with 100 μl of 1 mg/ml matrigel (dissolved in serum-free RPMI1640 medium). 600 μl of RPMI1640 medium containing 10% FBS was added to the lower chambers. Forty-eight hrs post-transfection, homogeneous single cell suspensions (1 × 10^5 ^cells/well) were added to the upper chambers and allowed to invade for 24 hrs at 37°C in a CO_2 _incubator. Cells remaining attached to the upper surface of the filters were carefully removed with cotton swabs. Migrated cells were stained with 0.1% crystal violet for 10 min at room temperature and examined by light microscopy. Quantification of migrated cells was performed according to published criteria [27].

### Tube formation assay

Fifty microliters of growth factor-reduced matrigel was polymerized on 96-well plates. HUVECs were serum starved in RPMI1640 medium for 2 hrs. The cells were suspended in RPMI1640 medium preconditioned with siRNA-transfected SGC-7901 cells, added to the matrigel-coated wells at the density of 5 × 10^4 ^cells/well, and incubated at 37°C for 18 hrs. Tube formation was visualized using a Leitz inverted microscope equipped with a Sony color digital DXC-S500 camera. Quantification of antiangiogenic activity was calculated by measuring the length of tube walls formed between discrete endothelial cells in each well relative to the control.

### Statistical analysis

Unless otherwise stated, all data were shown as mean ± standard error of the mean (SEM). Statistical significance (*P *< 0.05) was determined by *t *test or analysis of variance (ANOVA) followed by assessment of differences using SPSS 12.0 software (SPSS Inc., Chicago, IL).

## Results

### siRNA suppressed the HPA expression in gastric cancer cells

**Figure 1:**
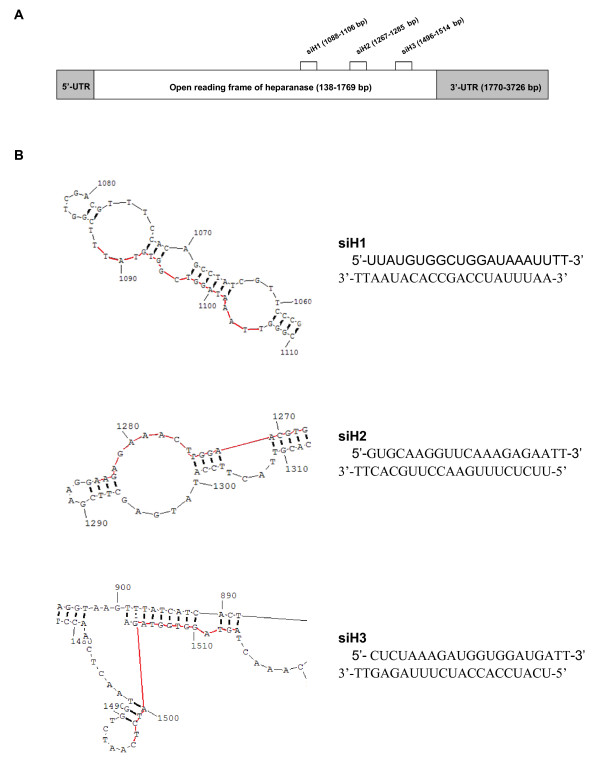
**Design of siRNA targeting different encoding regions of heparanase**. (A) Taken the heparanase mRNA (GenBank accession No. AF165154) as a target, three siRNA duplexes were designed and named as siH1, siH2, and siH3. Their corresponding targets located at 1088-1106 bp, 1267-1285 bp, and 1496-1514 bp of the encoding regions of heparanase mRNA. (B) Prediction of the secondary structures of siRNA targets was performed via RNAstructure 3.7 software. The siH1 recognized a region with three loops and two stems, while siH2 recognized a region with one loop and two stems. The target region of siH3 was sequestered intentionally in a stable stem structure. The red line indicated the target region of siRNA.

**Figure 2:**
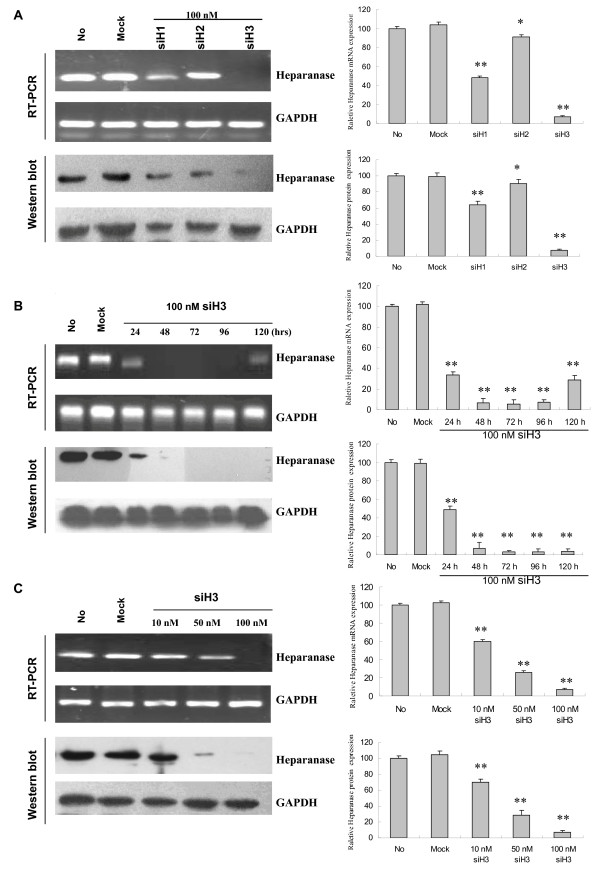
**siRNA suppressed the heparanase expression in gastric cancer cells**. Confluent gastric cancer SGC-7901 cells were seeded into 6-well plates, and transfected with different heparanase-specific siRNAs (siH1, siH2 and siH3) or scrambled siRNA (mock). The untransfected cells served as a control (No). The mRNA and protein expression of heparanase were examined by RT-PCR, real-time quantitative PCR and Western blot. (A) Forty-eight hrs post-transfection, siH3 (100 nmol/L) exerted the most efficiency in suppressing the heparanase expression, and siH1 (100 nmol/L) inhibited the heparanase expression to a lesser extent. However, transfection of siH2 (100 nmol/L) slightly influence the expression of heparanase in SGC-7901 cells. (B) The siH3 (100 nmol/L)-induced suppression of heparanase expression of SGC-7901 cells started at 24 hrs, and lasted for 120 hrs. Of note, the heparanase mRNA levels, but not the heparanase protein levels, were partially restored at 120 hrs post-transfection. (C) Transfection of different concentrations (10 nmol/L, 50 nmol/L and 100 nmol/L) of siH3 into SGC-7901 cells for 48 hrs, resulted in decrease of heparanase expression in a dose-dependent manner. The symbols (* and **) indicates a significant (*P *< 0.05) and a very significant (*P *< 0.01) decrease from mock, respectively. Triplicate experiments were performed with essentially identical results.

As shown in Figure 1A, three siRNA duplexes targeting different encoding regions of human HPA mRNA, named as siH1, siH2 and siH3, were designed and synthesized. Their siRNA sequences and the secondary structures of target mRNA were shown in Figure 1B. Under the mediation of Genesilencer, siRNAs were transfected into gastric cancer cell line SGC-7901. The mRNA and protein expression of HPA were examined by RT-PCR, real-time quantitative PCR and Western blot. As shown in Figure 2A, high HPA mRNA and protein levels were detected in the parental cells, and the scrambled siRNA control (mock, 100 nmol/L) did not affect the expression levels of HPA. The siH3 (100 nmol/L) exerted the most efficiency in suppressing the HPA expression, and siH1 (100 nmol/L) inhibited the HPA expression to a lesser extent (Figure 2A). However, transfection of siH2 (100 nmol/L) slightly influenced the expression of HPA in SGC-7901 cells (Figure 2A). In addition, the siH3-induced suppression of HPA expression of SGC-7901 cells started at 24 hrs, and lasted for 120 hrs (Figure 2B). Of note, the HPA mRNA levels, but not the HPA protein levels, were partially restored at 120 hrs post-transfection (Figure 2B). Moreover, transfection of different concentrations (10 nmol/L, 50 nmol/L and 100 nmol/L) of siH3 into SGC-7901 cells for 48 hrs, resulted in decrease of HPA expression in a dose-dependent manner (Figure 2C). These results indicated that the siRNAs, siH3 and siH1, were efficient in down-regulating the expression of HPA in gastric cancer cells.

### Silencing HPA attenuated the in vitro cell proliferation of gastric cancer cells

**Figure 3:**
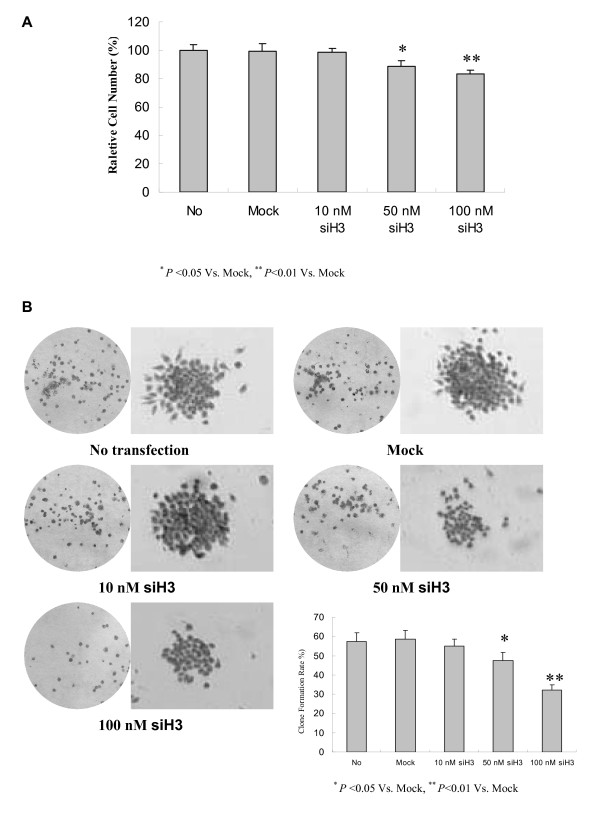
**Silencing heparanase attenuated the *in vitro *cell proliferation of gastric cancer cells**. Confluent SGC-7901 cells were seeded into 96-well plates, and transfected with different concentrations (10 nmol/L, 50 nmol/L, and 100 nmol/L) of siH3 or scrambled siRNA (mock, 100 nmol/L). The untransfected cells served as a control (No). (A) Forty-eight hours post-transfection, MTT colorimetry was performed to assay the cell proliferation. The results indicated that high concentrations of siH3 (50 nmo/L and 100 nmol/L), but not low concentration of siH3 (10 nmol/L) or scrambled siRNA (mock, 100 nmol/L), attenuated the proliferation of SGC-7901 cells, when compared to the parental cells. (B) In colony formation assay, 48 hrs post-transfection, the cells were seeded at a density of 300/ml on 35-mm dishes. Colonies were allowed to grow for 10-14 days. Positive colony formation (more than 50 cells/colony) was counted. The results indicated that high concentrations of siH3 (50 nmo/L and 100 nmol/L), but not low concentration of siH3 (10 nmol/L) or scrambled siRNA (mock, 100 nmol/L), attenuated the cellular colony formation rates of cultured SGC-7901 cells. The symbols (* and **) indicates a significant (*P *< 0.05) and a very significant (*P *< 0.01) decrease from mock, respectively. Triplicate experiments were performed with essentially identical results.

Since previous studies indicate that HPA modulates the cell proliferation of cancer cells [28], we first examined the effects of HPA siRNA on cell proliferation of SGC-7901 cells by MTT colorimetric assay. We found that transfection of siH3 for 48 hrs attenuated the cell proliferation in a dose-dependent manner, when compared to the parental cells and mock group (Figure 3A). In addition, the colony formation assay further revealed that 48 hrs post-transfection, high concentrations of siH3 (50 nmo/L and 100 nmol/L), but not low concentration of siH3 (10 nmol/L), attenuated the cell proliferation of cultured SGC-7901 cells (Figure 3B). These results indicated that siH3 attenuated the *in vitro *cell proliferation of gastric cancer cells.

### Silencing HPA abolished adhesion, migration and invasion of gastric cancer cells in vitro

**Figure 4:**
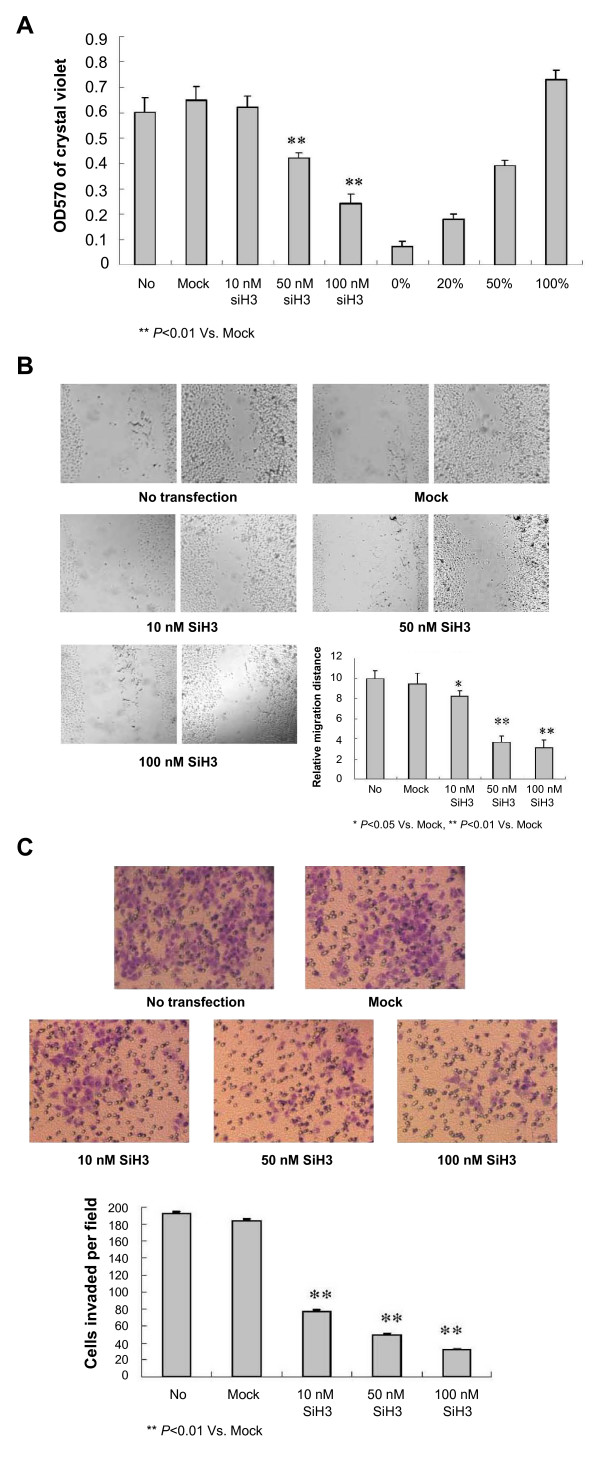
**Silencing heparanase abolished the adhesion, migration and invasion of gastric cancer cells *in vitro***. Confluent SGC-7901 cells were seeded into 96-well or 24-well plates, and transfected with different concentrations (10 nmol/L, 50 nmol/L, and 100 nmol/L) of siH3 or scrambled siRNA (mock, 100 nmol/L). The untransfected cells served as a control (No). (A) In the adhesion assay, 48 hrs post-transfection, 2 × 10^4 ^cells were inoculated into each matrigel-coated well of 96-well plates for 2 hrs, and washed three times with PBS. The results indicated that transfection of high concentrations of siH3 (50 nmo/L and 100 nmol/L), but not low concentration of siH3 (10 nmol/L) or mock, reduced the adhesion of SGC-7901 cells to the matrigel. (B) In scratch migration assay, 48 hrs post-transfection, the cells were scraped with 1-ml pipette tips (time 0). Cell migration into the wounded empty space was followed after 24 hrs and photographed. The results indicated that transfection of siH3 impaired the cellular migration in a dose-dependent manner. (C) In transwell analysis, 48 hrs post-transfection, homogeneous single cell suspensions (1 × 10^5 ^cells/well) were added to the upper chambers and allowed to invade for 24 hrs. Migrated cells were stained with 0.1% crystal violet and examined by light microscopy. The results indicated that transfection of siH3 abolished the invasion of SGC-7901 cells in a dose-dependent manner. The symbols (* and **) indicates a significant (*P *< 0.05) and a very significant (*P *< 0.01) decrease from mock, respectively. Triplicate experiments were performed with essentially identical results.

Since HPA plays critical roles in invasion and metastasis of cancer cells [9], and three critical steps are involved in metastasis, that is adhesion, migration and invasion [9], we further observed the effects of HPA-specific siH3 on these characteristics of SGC-7901 cells. In the adhesion assay, 48 hrs post-transfection, high concentrations of siH3 (50 nmo/L and 100 nmol/L), but not low concentration of siH3 (10 nmol/L), reduced the ability of SGC-7901 cells in adhesion to the precoated matrigel, when compared to parental cells (Figure 4A). However, the cells transfected with scrambled siRNA control (mock, 100 nmol/L) had similar ability in adhesion as parental cells (Figure 4A). In addition, 48 hrs post-transfection, the cells transfected with siH3 demonstrated an impaired migration capacity in a dose-dependent manner, when compared to the parental cells and mock group as evidenced by scratch migration assay (Figure 4B). Moreover, the transwell analysis indicated that transfection of siH3 for 48 hrs abolished the invasive capabilities of SGC-7901 cells in a dose-dependent manner, when compared to the parental cells and mock group (100 nmol/L) (Figure 4C). These results suggested that HPA-specific siH3 suppressed the adhesion, invasion and metastasis of gastric cancer cells *in vitro*.

### Silencing HPA inhibited the in vitro angiogenesis of gastric cancer cells

**Figure 5:**
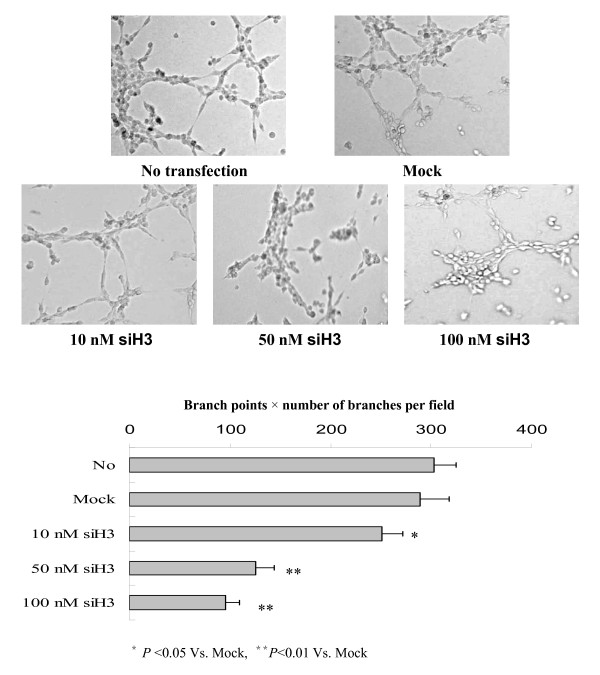
**Silencing heparanase inhibited the *in vitro *angiogenesis of gastric cancer cells**. Fifty microliters of growth factor-reduced matrigel was polymerized on 96-well plates. HUVECs were serum starved in RPMI1640 medium for 2 hrs, suspended in RPMI1640 medium preconditioned with siH3-transfected SGC-7901 cells, added to the matrigel-coated wells at the density of 5 × 10^4 ^cells/well, and incubated at 37°C for 18 hrs. Tube formation was visualized and calculated by measuring the length of tube walls formed between discrete endothelial cells in each well. Extensive tube formation of endothelial cells was observed in non-transfection (No) and scrambled siRNA (mock, 100 nmol/L) groups. However, when the endothelial cells were treated by the medium preconditioned with siH3-transfected SGC-7901 cells, the tube formation was dose-dependently suppressed. The symbols (* and **) indicates a significant (*P *< 0.05) and a very significant (*P *< 0.01) decrease from mock, respectively. Triplicate experiments were performed with essentially identical results.

Since HPA participates in the angiogenesis of tumor [29,30], we further investigated the effects of HPA-specific siH3 on the *in vitro *angiogenesis capabilities of SGC-7901 cells. As shown in Figure 5, extensive tube formation of endothelial cells was observed in non-transfection and mock (100 nmol/L) groups. However, when the endothelial cells were treated by the medium preconditioned with siH3-transfected SGC-7901 cells, the tube formation was dose-dependently suppressed (Figure 5). These results indicate that transfection of siH3 remarkably decreased the angiogenesis of gastric cancer cells *in vitro*.

## Discussion

Degradation of basement membrane (BM) and extracellular matrix (ECM) comprises an initial and essential step for cancer cells to invade surrounding tissue and metastasize to distant organs [31]. Both the BM and ECM contain heparan sulfite (HS) and heparan sulfate proteoglycans (HSPGs) as major structural components, which are substrates for heparanase (HPA) [9]. Human HPA gene is located on chromosome 4q21.3, and its cDNA contains an open reading frame of 1629-bp that encodes for a 61.2 kDa polypeptide of 543 amino acids [32-34]. Preferential expression of HPA mRNA and protein in tumors is evident in tissue specimens derived from oral squamous cell carcinoma [35], hepatocellular carcinoma [36], and carcinomas of prostate [15], bladder [14], and pancreas [37]. It has been reported that HPA correlated with the metastatic potential of mouse B16 melanoma and Eb lymphoma cells [38]. Subcutaneous inoculation of non-metastatic Eb lymphoma cells engineered to over-express HPA resulted in a significant decease in survival time of the mice due to a massive liver infiltration [32]. These findings support the correlation between HPA expression and the metastatic capacity of cancer cells.

Since HPA plays an important role in the invasion and metastasis of cancer cells, many studies focus on the development of HPA inhibitors [23]. With the availability of recombinant HPA and the establishment of high-throughput screening methods, a variety of inhibitory molecules have been developed, including neutralizing antibodies, peptides, small molecules, modified non-anticoagulant species of heparin [23], as well as several other polyanionic molecules, such as laminaran sulfate, suramin and PI-88 [20,39,40]. These inhibitors that decrease HPA expression in cancer cells significantly reduce their metastatic properties, signifying the importance of HPA in cancer cell spread [20,23,39,40]. However, because of the multiple biologic activities of these compounds, the mechanism of their antitumor activity and its relation to HPA inhibition are not straightforward. Moreover, the pleiotropic interactions of these compounds with the ECM and the cell surface might produce nonspecific and undesirable effects [20,23,39,40]. Thus, novel approaches are needed to inhibit the role of HPA in cancer progression [23].

Recent report indicated that short hairpin RNA (shRNA) targeting mouse HPA reduced the metastatic potential of B16-BL6 mouse melanoma cells [38]. Mice inoculated with Eb lymphoma cells transfected with anti-HPA ribozyme exhibited a marked decrease in liver metastasis and survived significantly longer [38]. Current selection of efficient siRNAs relies mainly on the analysis of sequence elements that mediate efficient incorporation into the RNA-induced silencing complex (RISC) [41,42]. However, several studies have suggested the importance of target secondary structure and accessibility based on computational modeling of target structure [43-45]. In this study, we designed three siRNAs targeting the HPA, named as siH1, siH2 and siH3, according to the guidelines for siRNA design [46,47]. It is well established that once siRNAs are transfected into cells, the guide (antisense) strand of siRNA duplex is incorporated into the RISC, which identifies target mRNA that is complementary to the guide stranded [41,42]. An endoribonuclease then cleaves target mRNA, resulting in knock-down of gene expression [41,42]. We found that siH3 was most potent in suppressing the HPA expression in gastric cancer cells even through its predicted secondary structure of target mRNA was sequestered intentionally in a stable stem structure. These results were consistent with previous findings that large variation in the efficiency of siRNAs for different sites on the same target is commonly observed [48]. Thus, it is usually recommended to test several siRNAs in order to achieve the most potent one.

The relationship between HPA and cell proliferation is not established yet [49]. In this study, we found that down-regulation of HPA inhibited the proliferation of SGC-7901 cells *in vitro*. In addition, the biologic and therapeutic relevance of the HPA-silencing approach was validated using the cell adhesion assay, scratch migration assay, and matrigel assay. We found that the invasion and metastasis of gastric cancer cells *in vitro *were attenuated by HPA-specific siRNA. Apart from its involvement in invasion and metastasis of cancer, HPA is tightly involved in angiogenesis, primarily by means of releasing heparin-binding angiogenic factors sequestered by HS in BM and ECM, such as basic fibroblast growth factor, vascular endothelial growth factor, keratinocyte growth factor and hepatocyte growth factor [29,30]. A critical early event in the angiogenic process is degradation of the subendothelial BM, followed by endothelial cell migration toward the angiogenic stimulus [29,30]. By releasing HS-bound angiogenic growth factors from the ECM, active HPA may indirectly facilitate EC migration and proliferation [29,30]. In this study, we found that the angiogenesis of gastric cancer cells *in vitro *was suppressed by HPA-specific siRNA. However, we noted that even after knock-down of HPA, the gastric cancer cells still possessed the capabilities of invasion, metastasis, and angiogenesis. We believe that other factors, such as uPA and MMPs [4-7], also influence these characteristics of gastric cancer, which warrants our further investigation. The data described here indicate the potential application of HPA-specific siRNA in the treatment of gastric cancer. Since previous studies indicate that the intratumoral injection of siRNAs is a feasible and convenient method for preliminary evaluation of siRNA effect in animal models [50], further *in vivo *study is warranted to better evaluate the efficiency of HPA-specific siRNAs on the invasion and metastasis of gastric cancer.

## Conclusion

In summary, we have demonstrated that silencing the expression of human HPA can efficiently inhibit the invasion, metastasis and angiogenesis of human gastric cancer cells. It is likely that the inhibition of HPA expression possibly depresses the degrading of ECM and BM, thus inhibiting the invasion and metastasis of gastric cancer. Therefore, our study suggests that HPA-specific siRNAs may be of potential values as novel therapeutic agents for human gastric cancer.

## Competing interests

The authors declare that they have no competing interests.

## Authors' contributions

TQS designed the study, analyzed and interpreted the data, and drafted the manuscript. ZLD, JGS, MH and PJR performed cell culture, gene transfection and experimental detection. DJH and HXH were engaged in drafting the manuscript and in statistical analysis. All authors read and approved the final manuscript.

## Pre-publication history

The pre-publication history for this paper can be accessed here:

http://www.biomedcentral.com/1471-2407/10/33/prepub
